# Epetraborole pharmacokinetics/pharmacodynamics in the hollow fiber system model of *Mycobacterium tuberculosis*

**DOI:** 10.1128/aac.00481-25

**Published:** 2025-09-22

**Authors:** Sanjay Singh, Megan Devine, Tawanda Gumbo, Shashikant Srivastava

**Affiliations:** 1 Division of Infectious Diseases, Department of Medicine, University of Texas at Tyler School of Medicine 12347, Tyler, Texas, USA; 2 Department of Medicine, Section of Pulmonary and Critical Care, University of Texas at Tyler School of Medicine 12341, Tyler, Texas, USA; 3 Mathematical Modeling and AI Department, Praedicare Inc., Dallas, Texas, USA; 4 Hollow Fiber System and Experimental Therapeutics Laboratories, Praedicare Inc. 12341, Dallas, Texas, USA; 5 IMPI Biotech Consortium Inc., Tateguru, Zimbabwe; 6 Department of Cellular and Molecular Biology, The University of Texas Health Science Centre at Tyler, Tyler, Texas, USA; City St George's, University of London, London, United Kingdom

**Keywords:** *M. tuberculosis*, multidrug-resistance, epetraborole, pharmacokinetics, pharmacodynamics, clinical trial simulations

## Abstract

In the hollow fiber system model of tuberculosis (TB), the ratio of area under the concentration-time curve to MIC (AUC_0-24_/MIC) of 327.1 was identified as the epetraborole optimal exposure target for *Mycobacterium tuberculosis* kill. Monte Carlo simulation experiments showed that even the intravenous dose of 1,500 mg/twice daily would fail in the majority of patients, and the dose needed for good efficacy for TB may likely not be safe for patients.

## INTRODUCTION

Multidrug-resistant (MDR) tuberculosis (TB) has been declared a global emergency (1). The latest World Health Organization’s (WHO) global TB report estimated a total of 10.8 million people fell ill with TB in 2023, and an estimated 1.25 million died (2). The number of new antibiotics for clinical development to treat TB infections is limited, and the progress is further restricted by the high cost of drug development (3, 4). Therefore, the repurposing of drugs has been suggested to expedite the process to improve the treatment of drug-resistant TB (5). Here, we present the pharmacokinetics/pharmacodynamics (PK/PD) of epetraborole in the preclinical hollow fiber model system of TB (HFS-TB). Epetraborole is an oxaborole compound that inhibits bacterial leucyl transfer RNA synthetase and is currently undergoing clinical development with a focus on non-tuberculous mycobacteria (NTM), namely, *Mycobacterium avium* and *Mycobacterium abscessus* (3, 6). This study reports the minimum inhibitory concentration (MIC) distribution of epetraborole in drug-susceptible and drug-resistant clinical isolates of *Mycobacterium tuberculosis* (*Mtb*), a PK/PD study in the HFS-TB and *in silico* clinical trial simulations.

First, we performed MIC studies with 48 clinical isolates of *Mtb* (27 from Texas, USA, and 21 from Pretoria, South Africa). Twenty-six *Mtb* isolates were drug-susceptible, four were isoniazid mono-resistant, and 18 isolates were MDR-TB. The epetraborole MIC distribution for the 48 clinical isolates is shown in Table S1, where the MICs ranged between 0.25 mg/L and 64 mg/L, and the cumulative MIC for 50% of isolates (MIC_50_) was 2 mg/L, while the MIC_90_ was 32 mg/L. We observed that the epetraborole MICs were not affected by the pre-existing antimicrobial resistance to the first-line drugs.

Next, we performed an HFS-TB epetraborole PK/PD study using an extensively drug-resistant (XDR)-TB strain that had an epetraborole MIC of 0.5 mg/L. The epetraborole concentration-time profiles of six different doses in HFS-TB and model diagnostics are shown in Fig. S1A through C. The epetraborole clearance rate in the HFS-TB was 0.01 ± 0.00 L/h, the volume of distribution was 0.25 ± 0.04 L, and the half-life was 15.59 ± 1.72 h. The PK models were used to calculate the AUC_0-24_ achieved in each HFS-TB (equivalent to those achieved in the lung) and AUC_0-24_/MIC ratios (Table S2).

Figure S2A through H shows the total *Mtb* burden (as CFU/mL) and the epetraborole-resistant subpopulation versus time. All epetraborole exposures kept the total bacterial burden below stasis (day 0 or inoculum) for up to 14 days, after which resistance to epetraborole monotherapy emerged. The highest exposure, AUC_0-24_/MIC = 3258.46, had the least epetraborole-resistant subpopulation after 28 days of monotherapy.

**Fig 1:**
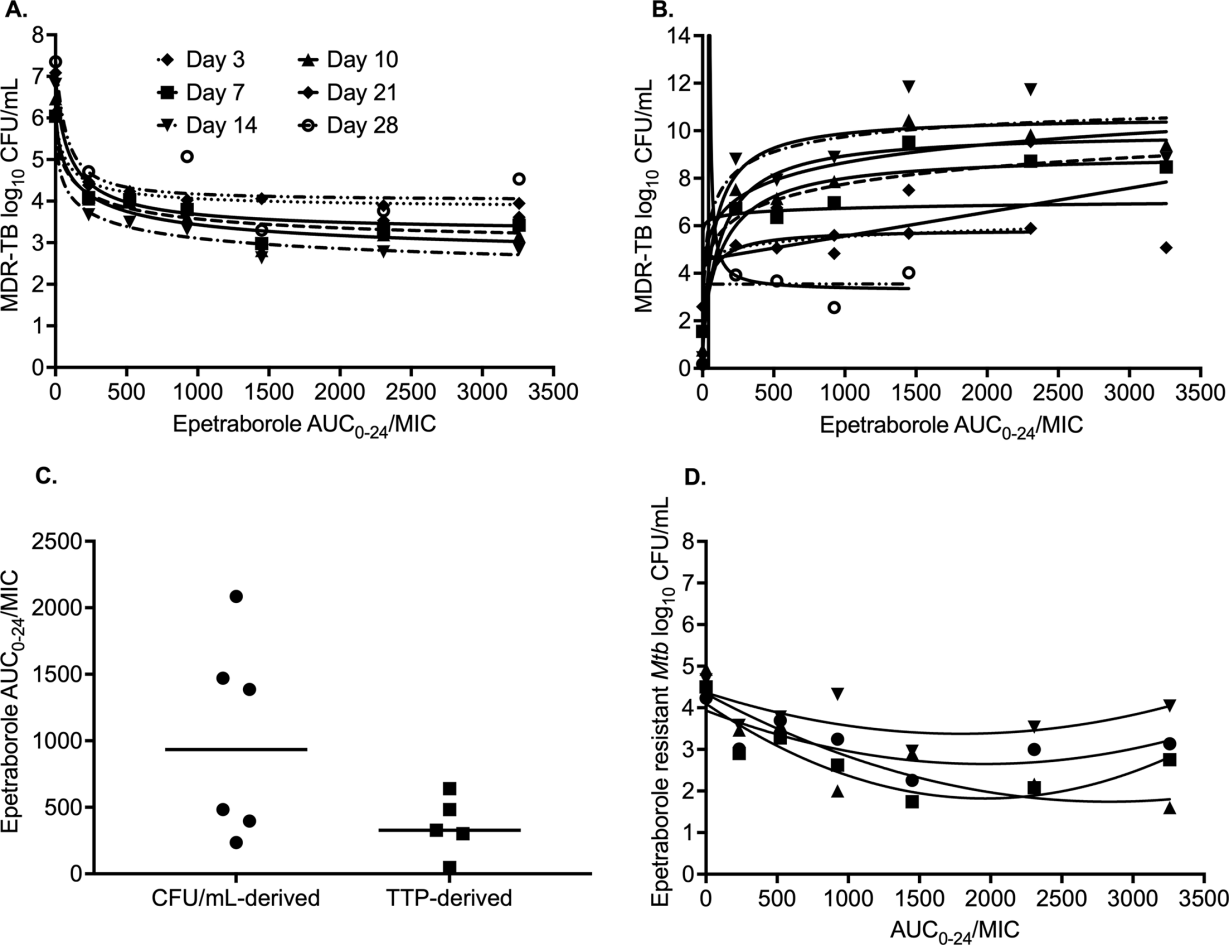
Epetraborole exposure-response relationship in the HFS-TB. (**A**) Curve fitting for each sampling day based on the CFU/mL readouts. (**B**) Curve fitting for each sampling day based on the MGIT-derived TTP readouts. (**C**) Comparison of EC_80_ values calculated using CFU/mL and TTP-derived readouts. (**D**) Exposure-dependent epetraborole antimicrobial resistance in the HFS-TB. (MGIT, Mycobacteria Growth Indicator Tube).

The relationship between epetraborole AUC_0–24_/MIC exposure and *Mtb* burden based on CFU/mL readout was modeled using the inhibitory sigmoid *E*_max_ model (Equation 1 in Supplemental methods)**,** with results shown in Fig. 1A and Table 1. Use of peak concentration (C_max_) to MIC and % of time concentration persisted above MIC (%T_MIC_) resulted in poorer corrected Akaike Information Criteria (AICc) score (7), although C_max_/MIC scores were similar in some instances because C_max_/MIC is co-linear with AUC/MIC. As regard the Mycobacteria Growth Indicator Tube liquid culture system-derived culture time-to-positive (TTP), model comparisons for inhibitory sigmoid *E*_max_ curve versus three-parameter agonist curves (Equation 2 in Supplemental methods) are shown in Table 1. Table 1 shows that the three-parameter model had better AICc scores each day than the four-parameter model, before even penalizing the four-parameter model for complexity. Therefore, the three-parameter model was chosen, with between-sampling day curves shown in Fig. 1B. The TTP-derived EC_80_ values were compared to those from CFU/mL readout in Fig. 1C. The median EC_80_ for TTP was an AUC_0-24_/MIC of 327.1 (95% confidence interval [CI]: 47.08–639.6) versus CFU-derived AUC_0-24_/MIC of 934.3 (95% CI: 235–2,065), *P* = 0.049.

**TABLE 1:** Summary of epetraborole PK/PD model parameter estimates across sampling days in the HFS-TB^*a*^

	Day 3	Day 7	Day 10	Day 14	Day 21	Day 28
Log_10_ CFU/mL Inhibitory Sigmoid *E*_max_ Model						
*E*_con_ (log_10_ CFU/mL)	6.12	6.04	6.46	6.81	7.08	7.35
*E*_max_ (log_10_ CFU/mL)	2.30	3.22	3.97	4.94	3.86	3.33
*H*	0.84	0.61	0.58	0.41	0.85	1.16
EC_50_ (mg*h/L)	76.02	142.8	134.7	70.23	94.49	71.13
Corrected AIC	24.43	49.10	48.77	44.58	52.86	60.65
*r*^2^	0.99	0.94	0.96	0.99	0.94	0.82
TTP Inhibitory Sigmoid *E*_max_ Model						
*E*_con_ (TTP)	2.60	1.56	0.78	0.45	No convergence	0.18
*E*_max_ (TTP)	−94.46	−12.30	−16.43	−11.17	No convergence	−6.53
*H*	0.13	0.33	0.27	0.53	No convergence	~3.19 × 10^−4^
EC_50_ (mg*h/L)	3.06 × 10^12^	957.00	1357.00	47.35	No convergence	~ 3.92^e−083^
Corrected AIC	Not estimated	64.93	65.33	72.84	No convergence	Not estimated
r^2^	Not estimated	0.92	0.94	0.88	No convergence	0.87
TTP 3 parameter agonist versus tesponse						
Bottom (TTP, days)	2.60	1.62	0.82	0.46	0.21	0.18
Top (TTP, days)	5.85	9.02	9.94	10.59	6.85	3.32
Span (TTP, days)	3.25	7.40	9.12	10.13	6.64	3.14
EC_50_ (mg*h/L)	81.78	159.90	123.20	75.37	11.77	0.00
Corrected AIC	26.83	23.92	24.60	31.03	33.22	26.83
*r*^2^	0.98	0.91	0.93	0.88	0.71	0.85
Quadratic function						
% Resistant-*Mtb* in non-treated controls		1.37		4.10	1.69	3.73
AUC_0–24_/MIC for resistance suppression		30,143.09		None	3,104.03	3,896.87
Corrected AIC		14.46		12.88	18.75	15.21
*r*^2^		0.90		0.44	0.52	0.78

^*a*^ TTP = time-to-positivity.

Next, modeling of AUC_0–24_/MIC versus effect for the drug-resistant CFU/mL, using the antibiotic resistance arrow of time, revealed the curves shown in Fig. 1D. The use of PK/PD parameters such as C_max_/MIC and %T_MIC_ resulted in poorer AICs and fits. Figure 1D shows a system of U-shaped curves that change with time, consistent with the antibiotic-resistance arrow of time in the HFS-TB and in the patients (8–10). Figure S3 could be more intuitive as it plots the resistant subpopulation as % of the total in each HFS-TB, with parameter estimates shown in Table 1. In Fig. S3, whereby amplification of resistance is defined as mediating a % of resistant subpopulation higher than in non-treated controls, the TTP-derived EC_80_ amplifies drug-resistance minimally to none at all, while the CFU-derived EC_80_ will amplify drug-resistance. The AUC/MIC shutting down all AMR is shown in Table 1.

**Fig 2:**
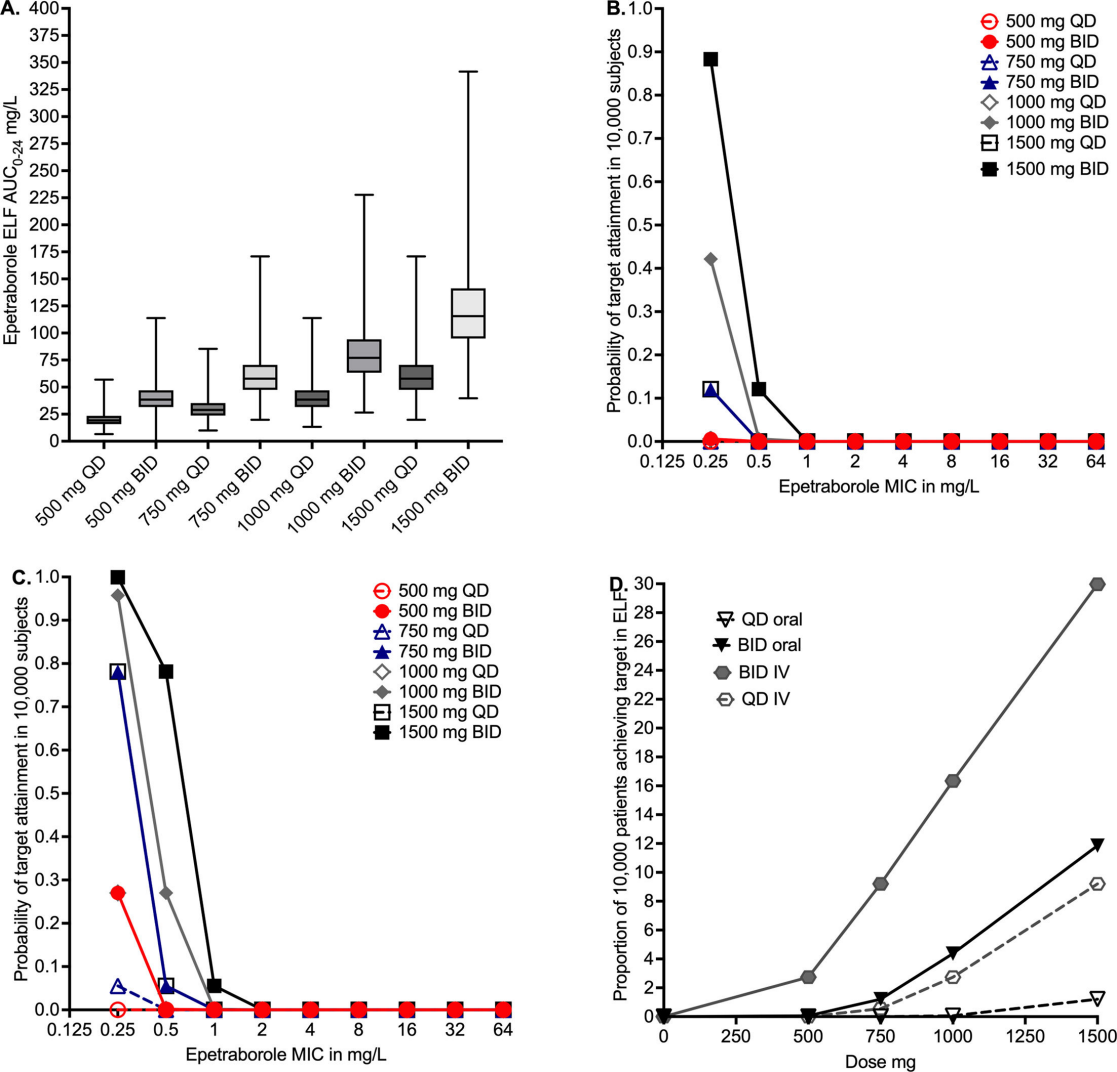
Monte Carlo experiments. (**A**) AUC_0–24_ with four different epetraborole doses with two administration routes, using population PK modeling. Notably, the AUCs with different doses overlap due to PK variability. This means that a dose-response study in patients would likely not yield conclusive results, highlighting the translational utility of the in silico approach. (**B**) None of the oral epetraborole doses, irrespective of the dosing schedule, showed >90% PTA. (**C**) Intravenous dosing showed better PTA compared to oral dosing. However, PTA for even the highest dose, 1,500 mg twice a day, falls below 90% at an MIC of 0.5 mg/L. (**D**) CFR for the different doses showing that none of the tested epetraborole doses achieved EC_80_ in >90% of patients.

Finally, we used the recently published Monte Carlo experiments (MCE) for epetraborole *in silico* dose-finding, using the population PK parameters based on Ganesan et al*.* (11) (S. Singh, G. D. Boorgula, M. H. Nguyen, et al., unpublished data). Figure 2A shows the AUC_0–24_ predicted to be achieved in the epithelial lining fluid (ELF) of doses with oral administration in 10,000 virtual subjects in the MCE. Figure 2B shows that none of the oral doses had a >90% probability of target attainment (PTA) even with twice daily dosing. Figure 2C shows that even with 1,500 mg twice daily intravenous dosing, PTA falls below 90% at an MIC of 0.5 mg/L. Figure 2D shows the cumulative fraction of response (CFR) for the different doses, where the highest intravenous dose achieved a CFR of only 30%.

In summary, we showed that epetraborole has efficacy against both drug-susceptible and MDR *Mtb*, and epetraborole exposures killed XDR-TB in the HFS-TB. However, monotherapy failed due to resistance emergence. The epetraborole PK/PD optimized exposure target for drug-resistant *Mtb* kill in the HFS-TB model was determined as AUC_0–24_/MIC of 934.3 for CFU/mL readout, while for TTP, readout was an AUC_0–24_/MIC of 327.1, and the MCE found that even a dose of 1,500 mg twice a day (i.e., 3,000 mg/day) would fail to achieve a PTA of >90% beyond epetraborole MIC of 0.25 mg/L. This means that most *Mtb* isolates were epetraborole-resistant using this PK/PD susceptibility breakpoint. Thus, the epetraborole exposures required for the therapeutic effect likely cannot be achieved with a safe clinical dose.

## Data Availability

The raw data for the results presented in the manuscript is available with the corresponding author, upon a reasonable request following UTHSCT’s data-sharing policy.

## SUPPLEMENTAL MATERIAL

Supplemental material aac.00481-25-s0001.pdf Supplemental methods; Tables S1 and S2; Fig. S1 to S3.

ASM does not own the copyrights to Supplemental Material that may be linked to, or accessed through, an article. The authors have granted ASM a non-exclusive, world-wide license to publish the Supplemental Material files. Please contact the corresponding author directly for reuse.
