# Supplementary material for: Epetraborole pharmacokinetics/pharmacodynamics in the hollow fiber system model of Mycobacterium tuberculosis
Source: Antimicrob Agents Chemother. 2025 Sep 22;69(11):e00481-25. doi: 10.1128/aac.00481-25 (PMC12587543; doi:10.1128/aac.00481-25)
Supplement: Supplemental material — Supplemental methods; Tables S1 and S2; Fig. S1 to S3. [file aac.00481-25-s0001.pdf]

**Epetraborole pharmacokinetics/pharmacodynamics in the hollow fiber system model of tuberculosis.**

Sanjay Singh[1], Megan Devine[2], Tawanda Gumbo[3,4,5], Shashikant Srivastava[1,6*]

[1]Division of Infectious Diseases, Department of Medicine, University of Texas at Tyler School of Medicine, Tyler, Texas, USA.

[2]Department of Medicine, Section of Pulmonary and Critical Care, University of Texas at Tyler School of Medicine, Tyler, Texas, USA.

[3]Mathematical Modeling and AI Department, Praedicare Inc., Dallas, Texas, USA.

[4]Hollow Fiber System & Experimental Therapeutics Laboratories, Praedicare Inc., Dallas, Texas, USA.

[5]IMPI Biotech Consortium Inc., Tateguru, Zimbabwe.

[6]Department of Cellular and Molecular Biology, The University of Texas Health Science Centre at Tyler, Tyler, Texas, USA.

[*]**Corresponding author:**

Shashikant Srivastava, Ph.D.

Division of Infectious Diseases, Department of Medicine,

University of Texas at Tyler School of Medicine,

11937 US Highway 271, Tyler, Texas, 75708, USA

Phone: (903) 877-7684

e-mail: Shashi.kant@uthct.edu

**Running title:** Epetraborole and *M. tuberculosis*.

## SUPPLEMENTARY METHODS

### Bacterial Isolates, drugs, and other supplies

We have a library of 48 clinical isolates of *Mtb* (27 from Texas, USA, and 21 from Pretoria, South Africa) that were used to generate epetraborole MIC distribution. Twenty-six isolates were drug-susceptible, four were isoniazid mono-resistant, and 18 isolates were MDR-TB. The HFS-TB study used one XDR-TB clinical isolate (SAMRC-16D); whole genome sequencing demonstrated mutations in *katG* (Ser315Thr), *rpoB* (Ser450Leu, Tyr564His), *embB* (Met306Val), *pncA* (Val139Gly), *gidB* (Leu16Arg, Ser100Phe), and *gyrA* (Clu21Gln, Ser95Thr, Gly247Ser, Gly668Asp) and ponA1 (Pro631Ser) (1, 2). These genes are associated with resistance to isoniazid, rifampin, ethambutol, pyrazinamide, aminoglycosides, fluoroquinolones, and β-lactams, respectively (3, 4). We used Middlebrook 7H9 broth supplemented with 10% oleic acid-albumin-catalase-dextrose (OADC), Middlebrook 7H10 agar supplemented with 10% OADC, and Middlebrook 7H9 broth supplemented with 2% dextrose in different experiments. Hollow-fiber cartridges (Cat#C7011) were purchased from FiberCell Systems Inc. (MD, USA). Epetraborole was purchased from BOC Sciences, NY, USA, and reconstituted in dimethyl sulfoxide (DMSO) followed by back dilution in sterile water. The final DMSO concentration was below 0.1% (v/v) having no effect on *Mtb* growth. The automated mycobacterial growth indicator tube (MGIT) liquid culture system, MGIT tubes, and Epicenter software were purchased from Becton Dickinson (Franklin Lakes, NJ, USA).

### Epetraborole minimum inhibitory concentration (MIC)

We used the broth microdilution method for MIC determination (5). Stock cultures were grown to logarithmic phase cultures. The inoculum was prepared by adjusting the turbidity to McFarland standard 0.5, followed by 100-fold dilution in normal saline to achieve a bacterial burden of ~$10^5$

CFU/mL. Next, 198 µL of the inoculum was added to each well of the sterile 96-well microtiter plate prefilled with 2 µL (100X) epetraborole solution. The epetraborole concentrations ranged between 0.125 to 64 mg/L. Plates were sealed in a Zip-lock bag, and cultures were incubated at 37°C. Starting 14 days of incubation, cultures were visually inspected daily until growth in nontreated controls was observed. The epetraborole concentration in the well that showed no visible growth, when the non-treated controls showed growth, was recorded as the MIC **(Table S1)**. The experiments were performed twice, with two replicates for each concentration.

**Epetraborole pharmacokinetics/pharmacodynamics (PK/PD) in HFS-TB**

The assembly and other details of HFS-TB have been published extensively in papers and the EMA regulatory approval documents (6-8). To determine the optimal exposure target of epetraborole for *Mtb* kill, logarithmic phase cultures of an XDR-TB clinical strain, SAMRC-16D, were used to prepare the inoculum as described above. Next, 20 mL of the inoculum was loaded into the peripheral compartment of each of the eight HFS-TB units. Two HFS-TB units served as non-treated controls, whereas the remaining six units were treated with different doses of epetraborole. The epetraborole peak concentrations ($C_{max}$) and AUCs with each of the six regimens are detailed in the results section below and **Table S2**. The drug was infused in the central compartment of each HFS-TB unit, except the nontreated controls, over 1 h ($T_{max}$) using a programmable syringe pump. The circulating medium in the HFS-TB was 7H9 broth supplemented with 2% dextrose (v/v) at pH 6.8. To capture the steady state concentration-time profile of epetraborole, each HFS-TB unit was sampled on study day seven at pre-dose (0), 1, 6, 11, 18, and 23.5 hours post-dose. Epetraborole concentration in each HFS-TB unit was measured using a validated stable-isotope dilution liquid chromatography-electrospray ionization tandem mass spectrometry (LC-ESI-MS/MS) method, published previously (9). To estimate the pharmacodynamics of bacterial growth in nontreated controls and kill with epetraborole, the

peripheral compartment of each HFS-TB unit was sampled on days 0, 3, 7, 10, 14, 21, and 28. The samples were washed twice using normal saline to remove residual epetraborole, followed by a 10-fold serial dilution. Processed samples were inoculated on 7H10 agar, and CFUs were recorded after 28 days of incubation at 37°C. As the second pharmacodynamic measure, 500 µl processed sample was added to MGIT tubes, and the time-to-positive (TTP) for each sample was recorded using the EpiCenter software. The time-in-incubation for the MGIT tubes was set to 56 days, and any tube that remained negative was considered a negative culture. To determine the epetraborole acquired antimicrobial resistance (AMR) during the treatment, processed samples were inoculated on 7H10 agar supplemented with epetraborole 3X MIC concentrations. The cultures were incubated up to 42 days at 37°C before CFUs were recorded.

**Pharmacokinetics/pharmacodynamics analyses**

We used ADAPT and WinNonLin for the PK analyses of the measured drug concentrations (10, 11). We employed a one-compartment PK model with zero-order input. PK parameters were estimated using the maximum-likelihood solution via the expectation-maximization algorithm (MLEM). The relationship between bacterial burden and epetraborole exposures (concentration or $AUC_{0-24}$/MIC) was modeled using the inhibitory sigmoid maximal effect ($E_{max}$) **Equation (1):**

$$\text{Effect } (\log_{10} \text{CFU/mL}) = E_{con} - E_{max} * EC^{H}/(EC^{H} + EC_{50}{}^{H}) \qquad \textbf{(1)}$$

$E_{con}$ in equation (1) is the bacterial burden in the non-treated controls, $EC_{50}$ is the concentration or exposure mediating 50% of $E_{max}$, and H is the Hill slope. The $EC_{80}$ was calculated and used as the PK/PD target for dose selection because this translates between the HFS-TB and patients, and estimation of optimal dose (12-18). Since TTP increases as bacterial burden declines, the dose response curve could be more akin to an agonist versus response three-parameter model in receptor theory:

$$\text{Effect (TTP; days)} = EC * \text{Span}/(EC + EC_{50}) \qquad \textbf{(2)}$$

We compared the TTP models **Equations (1)** and (**2**) based on Akaike Information Criteria (19), with a correction to avoid over-fitting. AMR modeling of bacterial burden versus exposure was modeled using Gumbo's antibiotic resistance arrow of time  quadratic function (20-22).

**Monte Carlo Experiments (MCE) for *in silico* dose finding.**

We have been using the MCE methods for *in silico* TB dose ranging using ADAPT 5 since 2004, with time-to-time updates as required (9, 14-16, 23-25). This is based on facts borne out in artificial intelligence (AI)-based findings we made ~15 years ago that PK variability, dose, and exposure-effect relationships drive account for >90% of TB therapeutic outcomes in patients (20, 26-33). In this regard, we recently published MCE methods for the epetraborole population PK model for *M. abscessus* cavitary disease based on Ganesan *et al* (9, 34). The serum PK parameter estimates (inter-individual variability as % coefficient of variation (%CV) from the three compartment model were total clearance of 15.3 L*h$^{-1}$(7.9%), central volume 1 (V1) of 15.6L (37.2%), clearance 2 of 23 L*h$^{-1}$ (30%), volume 2 of 140L (8.68%), clearance 3 of 43.3 L*h$^{-1}$ (30%), volume 3 of 33.2L (33.2%), and a 56.5% penetration into lung lesions (34)These values were used in the domain of input for the MCEs, in subroutine PRIOR of ADAPT 5. We exampled the ability of the following doses and dose schedules to achieve or exceed the $EC_{80}$ identified in the HFS-TB, in lung lesions: 500 mg once a day (QD), 500 mg twice a day (BID), 750 mg QD, 750 mg BID, 1000 mg QD, 1,000 mg BID, 1500 mg QD, and 1,500 mg BID. We then identified the probability of target attainment (PTA) at each MIC and then took an expectation for the entire MIC distribution for the cumulative fraction of response (17, 24).

## SUPPLEMENTARY RESULTS

The epetraborole concentration-time profiles, based on the measurement of the drug in each HFS-TB unit, are shown in **Supplementary Figure 1A**. The PK modeling calculated epetraborole

clearance rate in the HFS-TB was 0.01±0.00 L/h, volume of distribution as 0.25±0.04 L, and half-life of 15.59±1.72 h. The PK modeled versus predicted concentrations are shown in **Figure 1B**, where symbols on the y-axis at time zero represent residual epetraborole from the previous dosing cycle, due to a long half-life. None of the data was excluded from the analysis ($r^2 = 0.87$). Model diagnostics (residuals versus observation time) in **Figure 1C** show that there was minimal bias. The PK models were used to calculate the $AUC_{0-24}$ achieved in each HFS-TB (equivalent to those achieved in the lung), from which were calculated epetraborole $AUC_{0-24}$/MIC ratios **(Table S2)**.

**Figure S2A-G** shows the total *Mtb* burden (as CFU/mL) and the epetraborole-resistant subpopulation (as CFU/mL) versus time. **Figure S2H** shows the total *Mtb* burden as time-to-positivity (TTP) from the automated mycobacterial growth indicator tube (MGIT) liquid culture system. Based on CFU/mL readout, all epetraborole exposures kept the total bacterial burden below stasis (day 0 or inoculum) for up to 14 days, after which the trajectory changed, indicating the emergence of AMR in epetraborole monotherapy-treated HFS-TB units. The highest exposure, $AUC_{0-24}$/MIC=3258.46, had the least epetraborole-resistant subpopulation after 28 days of monotherapy. Based on TTP, all epetraborole exposures kept bacterial below day 0 burden ($B_0$), except AUC/MIC of 1449. The proportion of epetraborole-resistant *Mtb* in the nontreated control was 0.31±0.03% on day 28.

**Limitations of the study**

Our study has some limitations. First, HFS-TB is often criticized for performing the studies with a single isolate, as reported here with a single XDR-TB strain. Given that the treatment regimen for drug-susceptible TB performs well in a large proportion of patients, we excluded the possibility that epetraborole will be used for this subset of patients. Therefore, the studies were performed

with the XDR-TB clinical strain.  Second, there was only one HFS-TB unit per regimen, except for the nontreated controls. These proof-of-concept epetraborole PK./PD studies were thus not designed to account for the technical variability (17, 35). Third, we did not test epetraborole against different metabolic subpopulations of *Mtb*, namely semi-dormant bacilli growing under an acidic environment, intracellular *Mtb*, and nonreplicating persisters. Since the MCEs showed that epetraborole exposures required for *Mtb* kill cannot be achieved with safe doses, there was no point in performing such studies.

**Table S1. Epetraborole MIC distribution among 48 clinical isolates of *M. tuberculosis*.**

| Isolate ID | Origin | MIC (mg/L) | Susceptible/Resistant |
|---|---|---|---|
| SAMRC-16D | Pretoria, South Africa | 0.5 | XDR-TB |
| SAMRC-1A | Pretoria, South Africa | 0.25 | Susceptible |
| SAMRC-3A | Pretoria, South Africa | 0.25 | Susceptible |
| SAMRC-7A | Pretoria, South Africa | 0.5 | Susceptible |
| SAMRC-14A | Pretoria, South Africa | 8 | Susceptible |
| SAMRC-11B | Pretoria, South Africa | 2 | Susceptible |
| SAMRC-8A | Pretoria, South Africa | 2 | Susceptible |
| SAMRC-18B | Pretoria, South Africa | 8 | Susceptible |
| SAMRC-10B | Pretoria, South Africa | 16 | Susceptible |
| SAMRC-1C1 | Pretoria, South Africa | 2 | MDR-TB |
| SAMRC-3D3 | Pretoria, South Africa | 0.5 | MDR-TB |
| SAMRC-5D | Pretoria, South Africa | 2 | MDR-TB |
| SAMRC-9C | Pretoria, South Africa | 0.5 | MDR-TB |
| SAMRC-10C2 | Pretoria, South Africa | 0.5 | MDR-TB |
| SAMRC-12D4 | Pretoria, South Africa | 2 | MDR-TB |
| SAMRC-11D1 | Pretoria, South Africa | 4 | MDR-TB |
| SAMRC-18D3 | Pretoria, South Africa | 4 | MDR-TB |
| SAMRC-7C4 | Pretoria, South Africa | 2 | MDR-TB |
| SAMRC-2D | Pretoria, South Africa | 0.5 | MDR-TB |
| SAMRC-8C | Pretoria, South Africa | 2 | MDR-TB |
| SAMRC-6C | Pretoria, South Africa | 1 | MDR-TB |
| AMCC2308334 | Texas, USA | 2 | Susceptible |

| | | | |
|---|---|---|---|
| AMCC2309166 | Texas, USA | 0.5 | Isoniazid mono-resistant |
| AMCC2309406 | Texas, USA | 0.5 | MDR-TB |
| AMCC2310146 | Texas, USA | 16 | Isoniazid mono-resistant |
| AMCC2311648 | Texas, USA | 0.5 | Susceptible |
| AMCC2311733 | Texas, USA | 64 | MDR-TB |
| AMCC2312762 | Texas, USA | 16 | Susceptible |
| AMCC2313858 | Texas, USA | 32 | Susceptible |
| AMCC2313987 | Texas, USA | 32 | Susceptible |
| AMCC2315134 | Texas, USA | 0.5 | Susceptible |
| AMCC2316189 | Texas, USA | 0.5 | Susceptible |
| AMCC2316706 | Texas, USA | 64 | Susceptible |
| AMCC2401521 | Texas, USA | 32 | Susceptible |
| AMCC2402506 | Texas, USA | 32 | MDR-TB |
| AMCC2402772 | Texas, USA | 16 | Susceptible |
| AMCC2402988 | Texas, USA | 1 | Susceptible |
| AMCC2404114 | Texas, USA | 16 | Susceptible |
| AMCC2405161 | Texas, USA | 16 | Susceptible |
| AMCC2405570 | Texas, USA | 1 | Susceptible |
| AMCC2406524 | Texas, USA | 32 | MDR-TB |
| AMCC2409186 | Texas, USA | 0.25 | Susceptible |
| AMCC2411696 | Texas, USA | 0.5 | MDR-TB |
| AMCC2412511 | Texas, USA | 16 | Susceptible |
| AMCC2412589 | Texas, USA | 2 | MDR-TB |
| AMCC2413302 | Texas, USA | 0.25 | Susceptible |

| | | | |
|---|---|---|---|
| AMCC2413509 | Texas, USA | 0.25 | Isoniazid mono-resistant |
| AMCC2415484 | Texas, USA | 0.5 | Susceptible |

Susceptible, susceptible to isoniazid and rifampin, MDR-TB, simultaneous resistance to isoniazid and rifampin; XDR-TB, simultaneous resistance to isoniazid and rifampin plus second line drugs.

**Table S2. Regimen and associated epetraborole exposures achieved in the HFS-TB.**

| HFS-TB Unit ID | $AUC_{0-24}$ | $AUC_{0-24}$/MIC | $C_{max}$ | $C_{max}$/MIC |
|---|---|---|---|---|
| R1 | 116.25 | 232.5 | 6.519 | 13.038 |
| R2 | 260.79 | 521.58 | 16.63 | 33.26 |
| R3 | 462.32 | 924.64 | 31.05 | 62.1 |
| R4 | 724.6 | 1449.2 | 48.6 | 97.2 |
| R5 | 1153.2 | 2306.4 | 71.23 | 142.46 |
| R6 | 1629.23 | 3258.46 | 100 | 200 |

**Figure S1**. **Epetraborole pharmacokinetics in HFS-TB on study day seven. (A).** Concentration-time profiles. Symbol represents the measured epetraborole concentrations in HFS-TB to capture the steady-state concentration-time profile and the solid line represents model predicted concentrations. **(B)** Regression between model Predicted versus observed epetraborole concentrations in the HFS-TB for PK modelling. Since the pharmacokinetic sampling of the HFS-TB units was performed on study day seven to capture the steady-state concentration-time profile. The symbols on the y-axis at time zero represent residual epetraborole from previous dosing cycle, due to a long half-life. None of the data was excluded from the analysis. **(C)** Pharmacokinetic model diagnostics (residuals) for epetraborole concentration in each of HFS-TB unit to show a good model fit with minimal bias.

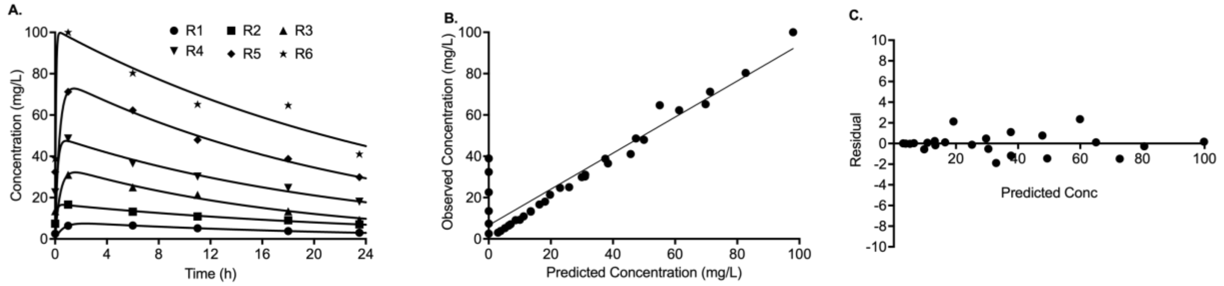

**Figure S2. Epetraborole time-kill and resistance curves in the HFS-TB over 28-day study duration.** The solid line indicates the total bacterial burden, and the dotted line represents epetraborole resistant subpopulation.

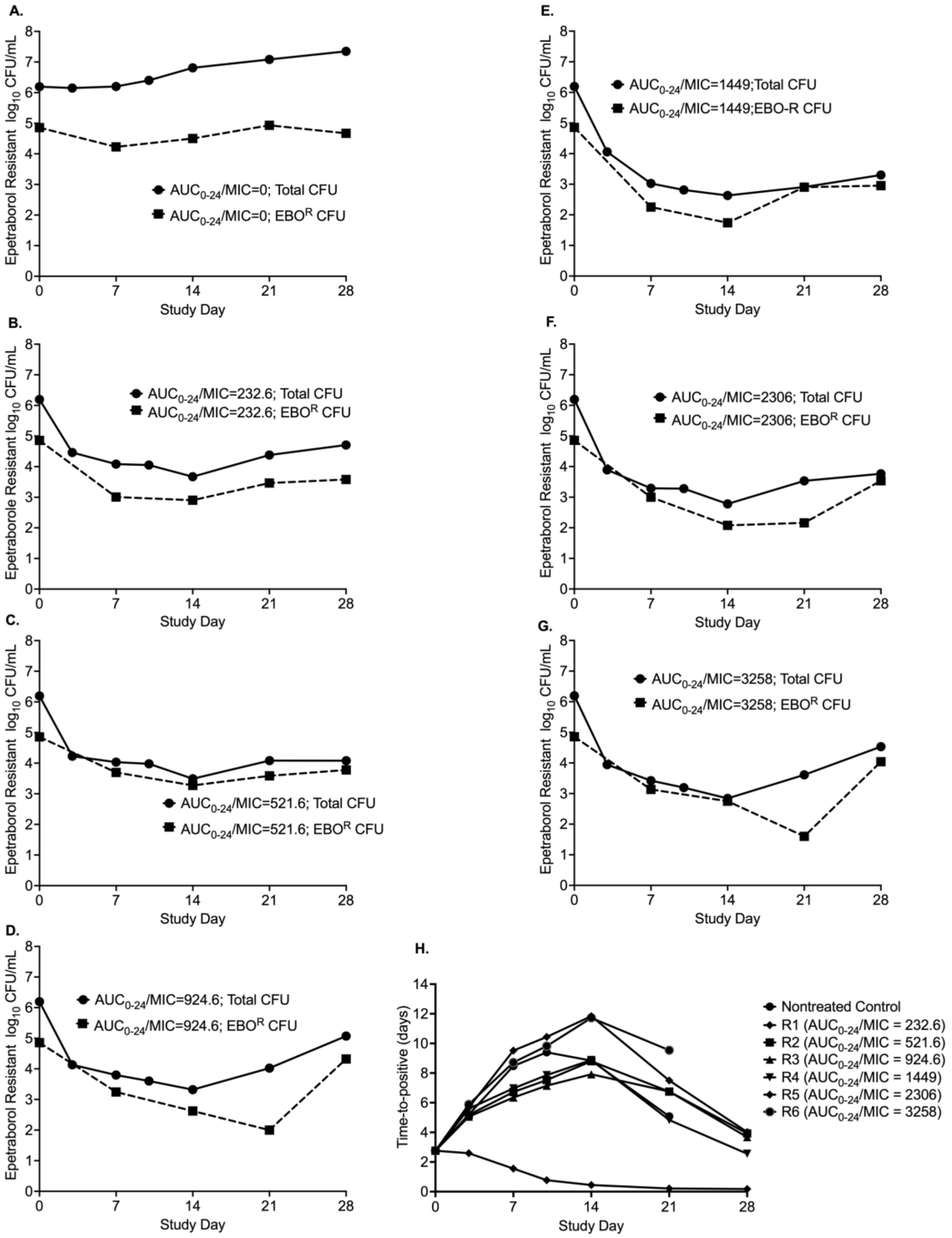

**Figure S3. Epetraborole-resistant subpopulation as % of the total in HFS-TB on different sampling days.** Amplification of resistance was defined as epetraborole exposure mediating a % of resistant subpopulation higher than in non-treated controls. It was observed that the CFU-derived $EC_{80}$ exposure will amplify antimicrobial resistance.

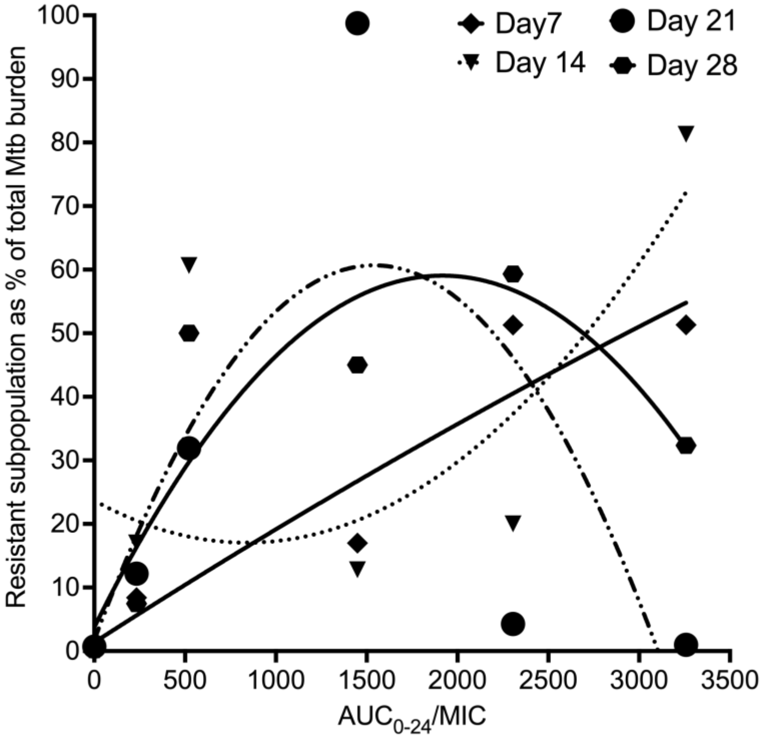

## REFERENCES


1. Srivastava S, Thomas T, Howe D, Malinga L, Raj P, Alffenaar JW, Gumbo T. 2021. Cefdinir and β-Lactamase Inhibitor Independent Efficacy Against Mycobacterium tuberculosis. Front Pharmacol 12:677005.
2. Singh S, Gumbo T, Boorgula GD, Thomas TA, Philley JV, Srivastava S. 2024. Omadacycline pharmacokinetics/pharmacodynamics and efficacy against multidrug-resistant Mycobacterium tuberculosis in the hollow fiber system model. Antimicrob Agents Chemother 68:e0108023.
3. Dheda K, Gumbo T, Maartens G, Dooley KE, McNerney R, Murray M, Furin J, Nardell EA, London L, Lessem E, Theron G, van Helden P, Niemann S, Merker M, Dowdy D, Van Rie A, Siu GK, Pasipanodya JG, Rodrigues C, Clark TG, Sirgel FA, Esmail A, Lin HH, Atre SR, Schaaf HS, Chang KC, Lange C, Nahid P, Udwadia ZF, Horsburgh CR, Jr., Churchyard GJ, Menzies D, Hesseling AC, Nuermberger E, McIlleron H, Fennelly KP, Goemaere E, Jaramillo E, Low M, Jara CM, Padayatchi N, Warren RM. 2017. The epidemiology, pathogenesis, transmission, diagnosis, and management of multidrug-resistant, extensively drug-resistant, and incurable tuberculosis. Lancet Respir Med S2213-2600:30079-6.
4. Deshpande D, Srivastava S, Chapagain M, Magombedze G, Martin KR, Cirrincione KN, Lee PS, Koeuth T, Dheda K, Gumbo T. 2017. Ceftazidime-avibactam has potent sterilizing activity against highly drug-resistant tuberculosis. Sci Adv 3:e1701102.
5. CLSI. 2018. *Susceptibility testing of mycobacteria, nocardia spp., and other aerobic actinomycetes*. 3rd ed. CLSI Standard M24.
6. Srivastava S, Gumbo T. 2011. *in vitro* and *in vivo* modeling of tuberculosis drugs and its impact on optimization of doses and regimens. Curr Pharm Des 17:2881-8.

7. Srivastava S, Pasipanodya JG, Meek C, Leff R, Gumbo T. 2011. Multidrug-resistant tuberculosis not due to noncompliance but to between-patient pharmacokinetic variability. J Infect Dis 204:1951-9.

8. Singh S, Gumbo T, Alffenaar JW, Boorgula GD, Shankar P, Thomas TA, Dheda K, Malinga L, Raj P, Aryal S, Srivastava S. 2023. Meropenem-vaborbactam restoration of first-line drug efficacy and comparison of meropenem-vaborbactam-moxifloxacin versus BPaL MDR-TB regimen. Int J Antimicrob Agents 62:106968.

9. Singh S, Boorgula GD, Nguyen MH, Daley CL, Gumbo T, Srivastava S. 2025. Epetraborol in pre-clinical models of *Mycobacterium abscessus* lung disease. J Antimicrob Chemother Manuscript # JAC-2024-1585.

10. D'Argenio DZ, Schumitzky A. 1997. ADAPT II. A program for simulation, identification, and optimal experimental design. User manual. Biomedical Simulations Resource, University of Southern California, Los Angeles, California, USA.

11. No Author Listed. 2020. Phoenix® WinNonlin® version 8.1 (Certara USA, Inc., Princeton, NJ),

12. Mallikaarjun S, Chapagain ML, Sasaki T, Hariguchi N, Deshpande D, Srivastava S, Berg A, Hirota K, Inoue Y, Matsumoto M, Hafkin J, Geiter L, Wang X, Gumbo T, Liu Y. 2020. Cumulative Fraction of Response for Once- and Twice-Daily Delamanid in Patients with Pulmonary Multidrug-Resistant Tuberculosis. Antimicrob Agents Chemother 65.

13. Liu Y, Moodley M, Pasipanodya JG, Gumbo T. 2023. Determining the Delamanid Pharmacokinetics/Pharmacodynamics Susceptibility Breakpoint Using Monte Carlo Experiments. Antimicrob Agents Chemother 67:e0140122.

14. Gumbo T, Pasipanodya JG, Nuermberger E, Romero K, Hanna D. 2015. Correlations between the hollow fiber model of tuberculosis and therapeutic events in tuberculosis patients: learn and confirm. Clin Infect Dis 61 Suppl 1:S18-24.

15. Pasipanodya JG, Nuermberger E, Romero K, Hanna D, Gumbo T. 2015. Systematic analysis of hollow fiber model of tuberculosis experiments. Clin Infect Dis 61 Suppl 1:S10-7.

16. Gumbo T, Pasipanodya JG, Romero K, Hanna D, Nuermberger E. 2015. Forecasting Accuracy of the Hollow Fiber Model of Tuberculosis for Clinical Therapeutic Outcomes. Clin Infect Dis 61 Suppl 1:S25-31.

17. Gumbo T, Angulo-Barturen I, Ferrer-Bazaga S. 2015. Pharmacokinetic-pharmacodynamic and dose-response relationships of antituberculosis drugs: recommendations and standards for industry and academia. J Infect Dis 211 Suppl 3:S96-S106.

18. Gumbo T, Lenaerts AJ, Hanna D, Romero K, Nuermberger E. 2015. Nonclinical models for antituberculosis drug development: a landscape analysis. J Infect Dis 211 Suppl 3:S83-95.

19. Akaike H. 1974. A new look at the statistical model identification. IEEE Transactions on Automatic Control 19:716-723.

20. Deshpande D, Pasipanodya JG, Mpagama SG, Srivastava S, Bendet P, Koeuth T, Lee PS, Heysell SK, Gumbo T. 2018. Ethionamide Pharmacokinetics/pharmacodynamics-derived dose, the role of MICs in clinical outcome, and the resistance arrow of time in multidrug-resistant tuberculosis. Clin Infect Dis 67:10.

21. Schmalstieg AM, Srivastava S, Belkaya S, Deshpande D, Meek C, Leff R, van Oers NS, Gumbo T. 2012. The antibiotic resistance arrow of time: efflux pump induction is a general first step in the evolution of mycobacterial drug resistance. Antimicrob Agents Chemother 56:10.

22. Gumbo T, Dona CS, Meek C, Leff R. 2009. Pharmacokinetics-pharmacodynamics of pyrazinamide in a novel *in vitro* model of tuberculosis for sterilizing effect: a paradigm for

faster assessment of new antituberculosis drugs. Antimicrob Agents Chemother 53:3197-204.

23. Gumbo T, Louie A, Deziel MR, Parsons LM, Salfinger M, Drusano GL. 2004. Selection of a moxifloxacin dose that suppresses drug resistance in *Mycobacterium tuberculosis*, by use of an *in vitro* pharmacodynamic infection model and mathematical modeling. J Infect Dis 190:1642-51.

24. Pasipanodya J, Gumbo T. 2011. An oracle: antituberculosis pharmacokinetics-pharmacodynamics, clinical correlation, and clinical trial simulations to predict the future. Antimicrob Agents Chemother 55:24-34.

25. Gumbo T. 2010. New susceptibility breakpoints for first-line antituberculosis drugs based on antimicrobial pharmacokinetic/pharmacodynamic science and population pharmacokinetic variability. Antimicrob Agents Chemother 54:1484-91.

26. Pasipanodya JG, McIlleron H, Burger A, Wash PA, Smith P, Gumbo T. 2013. Serum drug concentrations predictive of pulmonary tuberculosis outcomes. J Infect Dis 208:1464-73.

27. Chigutsa E, Pasipanodya JG, Visser ME, van Helden PD, Smith PJ, Sirgel FA, Gumbo T, McIlleron H. 2015. Impact of nonlinear interactions of pharmacokinetics and MICs on sputum bacillary kill rates as a marker of sterilizing effect in tuberculosis. Antimicrob Agents Chemother 59:38-45.

28. Rockwood N, Pasipanodya JG, Denti P, Sirgel F, Lesosky M, Gumbo T, Meintjes G, McIlleron H, Wilkinson RJ. 2017. Concentration-Dependent Antagonism and Culture Conversion in Pulmonary Tuberculosis. Clin Infect Dis 64:1350-1359.

29. Pasipanodya JG, Smythe W, Merle CS, Olliaro PL, Deshpande D, Magombedze G, McIlleron H, Gumbo T. 2018. Artificial intelligence-derived 3-Way Concentration-dependent Antagonism of Gatifloxacin, Pyrazinamide, and Rifampicin During Treatment of Pulmonary Tuberculosis. Clin Infect Dis 67:S284-S292.

30. Deshpande D, Pasipanodya JG, Srivastava S, Bendet P, Koeuth T, Bhavnani SM, Ambrose PG, Smythe W, McIlleron H, Thwaites G, Gumusboga M, Van Deun A, Gumbo T. 2018. Gatifloxacin pharmacokinetics/pharmacodynamics-based optimal dosing for pulmonary and meningeal multidrug-resistant tuberculosis. Clin Infect Dis 67:S274-S283.

31. Magombedze G, Pasipanodya JG, Srivastava S, Deshpande D, Visser ME, Chigutsa E, McIlleron H, Gumbo T. 2018. Transformation Morphisms and Time-to-Extinction Analysis That Map Therapy Duration From Preclinical Models to Patients With Tuberculosis: Translating From Apples to Oranges. Clin Infect Dis 67:S349-S358.

32. Swaminathan S, Pasipanodya JG, Ramachandran G, Hemanth Kumar AK, Srivastava S, Deshpande D, Nuermberger E, Gumbo T. 2016. Drug Concentration Thresholds Predictive of Therapy Failure and Death in Children With Tuberculosis: Bread Crumb Trails in Random Forests. Clin Infect Dis 63:S63-S74.

33. Srivastava S, Pasipanodya JG, Ramachandran G, Deshpande D, Shuford S, Crosswell HE, Cirrincione KN, Sherman CM, Swaminathan S, Gumbo T. 2016. A long-term co-perfused disseminated tuberculosis-3D liver hollow fiber model for both drug efficacy and hepatotoxicity in babies. EBioMedicine 6:126-138.

34. Ganesan H, Safir MC, Bhavnani SM, Krause KM, Rubino CM. 2022. Population Pharmacokinetic Model Development for Epetraborole and Mycobacterium avium Complex (MAC) Lung Disease Patients Using Data from Phase 1 and 2 Studies. Open Forum Infect Dis 9:ofac492.645.

35. Gumbo T, Srivastava S, Deshpande D, Pasipanodya JG, Berg A, Romero K, Hermann D, Hanna D. 2023. Hollow-fibre system model of tuberculosis reproducibility and performance specifications for best practice in drug and combination therapy development. J Antimicrob Chemother 78:953-964.

319
